# Virucidal Effects of Dielectric Barrier Discharge Plasma on Human Norovirus Infectivity in Fresh Oysters (*Crassostrea gigas*)

**DOI:** 10.3390/foods9121731

**Published:** 2020-11-25

**Authors:** Man-Seok Choi, Eun Bi Jeon, Ji Yoon Kim, Eun Ha Choi, Jun Sup Lim, Jinsung Choi, Kwang Soo Ha, Ji Young Kwon, Sang Hyeon Jeong, Shin Young Park

**Affiliations:** 1 Institute of Marine Industry, Gyeongsang National University, Tongyeong 53064, Korea; pyn358@naver.com (M.-S.C.); eunb61@naver.com (E.B.J.); yooonn22@naver.com (J.Y.K.); 2 Department of Seafood and Aquaculture Science, Gyeongsang National University, Tongyeong 53064, Korea; 3 Department of Electrical and Biological Physics, Plasma Bioscience Research Center, Kwangwoon University, Seoul 01987, Korea; ehchoi@kw.ac.kr (E.H.C.); junsub117@gmail.com (J.S.L.); neoled@kw.ac.kr (J.C.); 4 Southeast Sea Fisheries Research Institute, National Institute of Fisheries Science, Tongyeong 53085, Korea; ksha@korea.kr (K.S.H.); kjy3t3@korea.kr (J.Y.K.); jsh1826@korea.kr (S.H.J.)

**Keywords:** dielectric barrier discharge plasma, food safety, human norovirus, propidium monoazide, shellfish

## Abstract

This study investigates the effects of dielectric barrier discharge (DBD) plasma treatment (1.1 kV, 43 kHz, N_2_ 1.5 L/min, 10~60 min) on human norovirus (HuNoV) GII.4 infectivity in fresh oysters. HuNoV viability in oysters was assessed by using propidium monoazide (PMA) as a nucleic acid intercalating dye before performing a real-time reverse transcription–quantitative polymerase chain reaction (RT-qPCR). Additionally, the impact of the DBD plasma treatment on pH and Hunter colors was assessed. When DBD plasma was treated for 60 min, the HuNoV genomic titer reduction without PMA pretreatment was negligible (<1 log copy number/µL), whereas when PMA treatment was used, HuNoV titer was reduced to >1 log copy number/µL in just 30 min. D_1_ and D_2_-value of HuNoV infectivity were calculated as 36.5 and 73.0 min of the DBD plasma treatment, respectively, using the first-order kinetics model (R^2^ = 0.98). The pH and Hunter colors were not significantly different (*p* > 0.05) between the untreated and DBD-plasma-treated oysters. The results suggest that PMA/RT-qPCR could help distinguish HuNoV infectivity without negatively affecting oyster quality following >30 min treatment with DBD plasma. Moreover, the inactivation kinetics of nonthermal DBD plasma against HuNoV in fresh oysters might provide basic information for oyster processing and distribution.

## 1. Introduction

Human norovirus (HuNoV), a nonenveloped and single-stranded RNA virus, is a member of the Caliciviridae family. The first outbreak of HuNoV was discovered in the USA in Norwalk, Ohio, in 1968, and since then, HuNoV has been considered a significant cause of vomiting and acute gastroenteritis every year [1]. HuNoV causes acute gastroenteritis, and the main symptoms of infection include diarrhea, vomiting, nausea, and abdominal pain. HuNoV affects all ages, and HuNoV gastroenteritis heals naturally within 1 to 3 days. HuNoV outbreaks are related to interpersonal contact, airborne routes, and the consumption of contaminated foods, and 70–80% of all outbreaks are related to the GII.4 genotype [2]. Foodborne transmission of HuNoV includes fresh fruits and vegetables treated with contaminated sewage water during the production and consumption of contaminated shellfish from excrement discharged to the coastal area [3]. Oysters are correctly regarded as the primary vector of HuNoV compared with other types of shellfish; there are many reasons for this, but the biggest reason is that oysters are usually eaten raw or semiheated [4]. In particular, oysters are farmed in coasts and estuaries, which are susceptible to environmental pollution by humans, and because they are less mobile, noroviruses and bacteria can accumulate in the oyster’s tissue [5]. Oysters are distributed after a depuration process to ensure hygienic safety from HuNoV and bacteria. Simultaneously, applying the depuration process using the appropriate parameters and process controls, most bacteria are effectively removed in a relatively short time, such as 12 h for *Salmonella* and 15 to 20 h for *E. coli* [6,7]. However, in the case of norovirus, it takes an average of 19 days or more to reduce 1 log [8]. Therefore, oysters destined for human consumption should be subjected to an effective microbial reduction process technique against HuNoV that has minimal effect on the perceived quality [9].

Real-time quantitative PCR (RT-qPCR) has been effectively used to quantitatively detect HuNoV from potentially contaminated food such as oysters [10]. However, RT-qPCR detects both infectious and noninfectious HuNoV, which requires additional molecular treatment. Therefore, accurate detection of the infectious virus in foods is critical and necessary to assess the risk for foodborne outbreaks since only the infectious virus can lead to foodborne infection [11].

Propidium monoazide (PMA) has been used in previous studies to distinguish viable and nonviable bacteria by DNA genome in bacterial RT-qPCR analysis [11]. In addition, Parshionikar et al. [11] adopted a PMA pretreatment technique as an appropriate indirect method to estimate infectious and noninfectious cells of enteric viruses, including norovirus. PMA pretreatment combined with RT-qPCR analysis was shown to be capable of detecting selectively infectious murine norovirus (MNV), hepatitis A virus (HAV), rotavirus, poliovirus type 1, echovirus 7, coxsackievirus B5, and HuNoV in food and the environment [12,13,14]. Thus, PMA is considered an appropriate way to predict HuNoV viability.

The antimicrobial effects of plasma technology have been well established, as reflected by a body of recent studies. Plasma is the fourth state of matter (e.g., solid, liquid, gas), which is defined as an ionized gas where the gas gets more potent energy [15]. When the plasma is generated, ions and electrons are separated, and reactive species and ozone, having high chemical reactivity, are generated [16]. López et al. [17] noted that reactive oxygen species (ROS) and reactive nitrogen species (RNS) generated through gas ionization exert antimicrobial effects through direct and nonspecific attacks against microbial cellular envelopes and intracellular components (e.g., DNA, RNA, viral capsid). Previous studies have demonstrated that plasma treatment is effective against fungi, bacteria, and endospores of bacteria, as well as viruses such as HuNoV [17,18]. Aboubakr et al. [19] noted that ROS and RNS produced by plasma obstruct virus adhesion and entry into host cells, thereby oxidizing certain amino acids of the VP1 domain (N-terminal arm, shell domain, protrusion domain), damaging the capsid protein (e.g., VP1). In this regard, some studies have demonstrated the virucidal effects of plasma on foodborne viruses (e.g., MNV-1, HAV, HuNoV, adenovirus) [20,21]. In particular, DBD plasma is known for food disinfection and sterilization technology as it can treat wide areas and minimize the impact on food quality because it is a nonthermal technology and discharges at atmospheric pressure [15,18].

Nevertheless, to our knowledge, there have been few studies on the impact of DBD plasma on shellfish such as oysters, which are often cited as causes of HuNoV food infection. Since HuNoV can also bind to the digestive tissues, including the midgut and digestive diverticulum of oysters [22], we judged homogenates of oyster digestive tissues to be suitable for use in the HuNoV viability study. Therefore, this study’s objective is to characterize the effects of DBD plasma treatment time (10–60 min) on reducing potential HuNoV infectivity in fresh oysters using PMA coupled with RT-qPCR while also monitoring the effects on oyster quality.

## 2. Materials and Methods

### 2.1. Virus Stock Preparation

A stool sample was collected from a patient with gastroenteritis symptoms at the Gyeonggi Institute of Health and Environment (GIHE, Gyeonggido, Korea) in 2012. GIHE confirmed that the sample was infected with the HuNoV GII.4 genotype, and the specimen was sent to the Waterborne Virus Bank (WAVA, Seoul, Korea) in 2013; confirmation that this specimen was the HuNoV GII.4 Sydney variant was reported in a previous study [23]. The complete nucleotide sequence of the GII.4 Sydney variant was analyzed by Park et al. [24]. The HuNoV GII.4 variant in 500 μL of phosphate-buffered saline (PBS; pH 7.2) was purchased from WAVA, with a genomic titer of 6.50 log copy number/μL. The HuNoV GII.4 stock was transported to the laboratory in a dry-ice box and stored at −80 °C until use.

### 2.2. Oyster Sample Preparation and HuNoV Inoculation

Pacific oysters were purchased from a local seafood market (Tongyeong, Korea). Before inoculation with HuNov GII.4, 3 g of midgut gland samples were isolated, homogenized, and transferred to 15 mL conical tubes. Each experiment’s homogenates were inoculated with 30 μL of virus suspension (approximately 6.5 log copy number/μL). Oysters were tested for HuNoV according to the draft international standard ISO 15216-1 [25]. Samples were placed on a biological safety cabinet (CHC Lab Co. LTD., Daejeon, Korea) for 1 h to facilitate the attachment of HuNoV GII.4 to the samples.

### 2.3. DBD Plasma Treatment of HuNoV in Oyster

**Figure 1:**
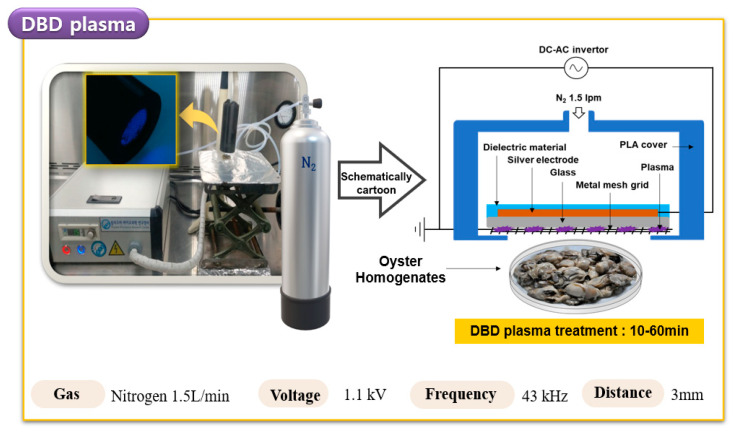
Schematic diagram of the dielectric barrier discharge (DBD) plasma system used for the oyster treatment. DC-AC, direct current and alternating current. PLA, polylactic acid.

The plasma equipment employed in this study is shown schematically in Figure 1. The DBD plasma device (μ-DBD Surface Plasma Generator, Model, Micro DBD plasma) was supplied by the Plasma Biomedicine Institute (Plasma Bioscience Research Center, Seoul, Korea) and has been described by Choi et al. [26]. The device was turned on for at least 10 min before the start of the experiment, and the surface of the oyster samples inoculated with HuNoV GII.4 was treated with DBD plasma for 0, 10, 20, 30, and 60 min in a sterile petri dish (35 × 15 mm). A distance of 3 mm was maintained between the plasma-emitting electrode and the sample during treatment. The silver electrode was deposited on the substrate glass plate using a screen-printing method, where thicknesses of the electrode and substrate glass were 10 μm and 1.8 mm, respectively. Dielectric material consisting of SiO_2_ was also screen-printed to 100 μm thickness.

A meshed metal grid was attached to the rear side of the glass and used as a grounded electrode. Gas flow can be guided to mesh surfaces by a polylactic acid cover via a gas injection hole. DBD plasma was generated on the rear glass surface between the glass and the meshed metal grid using a nitrogen flow rate of 1.5 L per minute (Lpm). DBD plasma, under a driving frequency of 43 kHz, indicated voltage and current characteristics, with a low discharge voltage of approximately 1 kV and a discharge peak current of 40 mA, respectively. The minimum discharge voltage for plasma production by DBD plasma devices used in this experiment was 1.1 kV. An air cooler (COOLERTEC Ice Bridge-1, COOLERTEC, Seoul, Korea) was also attached to the dielectric to prevent thermal effects when the plasma was continuously produced. After measuring the temperature at the time of plasma generation for up to 30 min, it was confirmed that the temperature range was 18.5~35 °C.

### 2.4. Propidium Monoazide Treatment on HuNoV in Oyster

HuNoV detection in oysters using PMA pretreatment was performed using the method described in Jeong et al. [27]. For dye treatment, treated virus samples (each 200 µL) were immediately mixed with 200 µM PMA (Biotium, Hayward, CA, USA). These mixtures were left in the dark at room temperature for 10 min to allow dye penetration. After that, the samples were exposed to 40 W LED light (Dynebio, Seongnam, Korea) at a 460 nm wavelength at room temperature for 20 min to photoactivate both dyes. HuNoV with damaged capsids and HuNoV with intact capsids were distinguished for HuNoV infectivity through PMA pretreatment. HuNoV infection samples without PMA pretreatment were set as control samples.

### 2.5. HuNoV Isolation and RNA Extraction

Samples of oyster homogenates sufficiently infected with HuNoV GII.4 were treated with the proteinase K (PK) method of Jeon et al. [14], revising the method of ISO 15216-1:2017 [25]. PK solution (100 μg/mL; Sigma-Aldrich, Dorset, UK) was added to 3 g of oyster homogenates in the same volume prepared at a final concentration of 0.1 mg/mL. After that, the sample was incubated at 37 °C by shaking at 290 rpm for 1 h. The sample was then incubated at 60 °C for 15 min, followed by centrifugation at 10,000× *g* (5400 rpm) for 10 min (SUPRA22K, Hanil Science Industrial Co., Gimpo, Korea), and the pellet was discarded. The extracted supernatant (approximately 4.0 mL) was collected in a sterilized 15 mL tube and stored at −80 °C until used for RNA extraction. Viral RNA was extracted and purified using the RNeasy Mini Kit (Qiagen, Hilden, Germany) in a final volume of 60 µL, according to the manufacturer’s protocol. The sample’s RNA was immediately subjected to RT-qPCR analysis to detect and quantify HuNoV GII.4 after extraction.

### 2.6. Quantitative Analysis of HuNoV Using RT-qPCR

For cDNA synthesis, reverse transcription was performed as in Kageyama et al. [28]. To amplify the HuNoV GII.4 gene, 1 µL of enzyme mix (5 units/µL), 5 µL of 5X RT-PCR buffer, 1 µL of 10 mM dNTP, 0.25 µL of RNase inhibitor (5 units/µL), 1 µL of 10 µM primer (forward and reverse), 5 μL of extracted RNA, and RNase-free water were added to a 25-μL final volume. RT-qPCR amplification was performed using a TP800-Thermal Cycler Dice Real-Time System (TaKaRa) as follows: initial denaturation at 95 °C for 10 min by 45 cycles of amplification at 53 °C for 25 s and 62 °C for 70 s. For HuNoV quantification, plasmid DNA (Takara, Korea), prepared by inserting the sequence (98 bp) of the HuNoV GII gene into the pET30a vector, was used. The RNA transcripts’ serial dilutions resulted in linear RT-qPCR standard curves, with a slope of −3.315 and a coefficient of determination (R^2^) > 0.99. Primers and probes were optimized for the ORF1- and ORF2-overlapping regions to increase sensitivity and specificity. The forward- and reverse primer sequences were COG2F: 5′-CAR GAR BCN ATG TTY AGR TGG ATG AG–3′ and COG2R: 5′-TCG ACG CCA TCT TCA TTC ACA-3′, respectively. The TaqMan probe was RING2: 5′-TGG GAG GGC GAT CGC AAT CT-3′, marked with the reporter fluorophore 5′-FAM and the quencher fluorophore 3′-TAMRA. HuNoV RNA (TaKaRa, Shiga, Japan) was used as a positive control, and RNase free water was used as a negative control.

### 2.7. DBD Plasma Reduction Kinetics

The reduction kinetics of microbial contaminants in oyster samples after DBD plasma treatment were analyzed for D-values (decimal reduction time) using a first-order kinetics model. The D-value calculation for HuNoV genomic titer reduction in the oyster samples followed the method described in Choi et al. [26].

$$log\frac{N}{N_{0}}\;=\;-\;\frac{k\;\cdot \;t}{2.303}=-\frac{1}{D}\times t$$

Here, *Nt* is the HuNoV GII.4 titer (log copy number/μL) at DBD plasma treatment time *t*, *N*_0_ is the initial HuNoV GII.4 titer (log copy/μL), and k is the reduction rate constant. Hence, it can be characterized by a single rate constant “k” or its reciprocal, the D_R_-value, which provides a quantitative measure of resistance to an applied lethal agent. D_R_-values refer to decimal reduction time. Thus, they can be used to calculate D_1_ and D_2_-values using the equation D_1_ = 2.303/k and D_2_ = 4.606/k.

### 2.8. Measurement of pH and Hunter Color

The whole oyster sample’s pH and Hunter colors were measured according to DBD plasma treatment time. The pH and Hunter colors were analyzed by the methods described in Choi et al. [26] and Albertos et al. [29]. For Hunter color measurement, the color of each whole oyster meat sample treated with DBD plasma was measured using a colorimeter (UltraScan PRO, Hunter Lab, Reston, VA, USA). The colorimeter was standardized using a calibration plate. A D_65_ illuminant and a 6-mm aperture were used for the colorimeter. The color parameters were represented by three coordinate values for each Hunter color: “L” from black (0) to white (100), “a” from green (-) to red (+), and “b” from blue (-) to yellow (+). For pH measurement, 10 g of each whole oyster meat sample was placed in a separate sterile sample bag (3M Korea, Seoul, Korea) along with 90 mL of distilled deionized water. After that, samples were homogenized for 3 min using a stomacher (Bagmixer, Interscience, Troy, MI, USA). The pH was then measured using a YSI 63 pH meter (Orion star A211, Thermo Scientific, Waltham, MI, USA).

### 2.9. Statistical Analysis

All experiments were carried out in triplicate, and results are presented as the mean and standard deviation. Results were subjected to a one-way analysis of variance (ANOVA) using statistical package for the social sciences (SPSS) version 25.0 application software (SPSS Inc., Chicago, IL, USA). HuNoV GII.4 samples, with and without PMA treatment and expressed in terms of logarithmic functions, pH, and Hunter color analysis, were analyzed; Duncan’s multiple range test was used to compare the differences in mean values. The paired *t*-test was carried out to evaluate the statistical significance of differences between PMA-treated and PMA-untreated samples using SPSS software. The means represented the average of three replicate samples, and they were considered significantly different at *p* < 0.05. Additionally, D_1_-and D_2_-values of HuNoV GII.4 in PMA-treated samples from raw oysters, following exposure to DBD plasma for different lengths of times, were also analyzed with a paired *t*-test using SPSS software. Model and parameter adequacies were considered significant at *p* < 0.05 unless otherwise noted.

## 3. Results

### 3.1. Influence of DBD Plasma Treatment on HuNoV GII.4 in Oysters by PMA/RT-qPCR

The oyster homogenates inoculated with HuNoV GII.4 were treated with DBD plasma for 0, 10, 20, 30, and 60 min to evaluate the viral activity of the DBD plasma treatment against HuNoV GII.4 in oysters. Without DBD plasma treatment, the initial HuNoV titer recovered from oyster homogenates was 5.51 copy number/μL; the recovery rate was 84.77% [(5.51/6.50 × 100)]. The results of HuNoV in oysters were largely explained in two parts. First, the results of DBD plasma treatment and the PMA pretreatment effect on HuNoV GII.4 were quantified using RT-qPCR (Table 1). The inoculated HuNoV GII.4 log titer in oysters was significantly decreased, with a stepwise increase in DBD plasma treatment time in both the non-PMA- and PMA-treated samples (*p* < 0.05). In the non-PMA-treated oysters, HuNoV GII.4 log reduction was increased, with a stepwise increase in DBD plasma treatment time as follows: 0.28 (10 min treatment), 0.40 (20 min treatment), 0.54 (30 min treatment), and 0.76 (60 min treatment). In the PMA-treated oysters, HuNoV GII.4 log reduction was increased, with a stepwise increase in DBD plasma treatment time as follows: 0.43 (10 min treatment), 0.68 (20 min treatment), 1.05 (30 min treatment), and 1.68 (60 min treatment).

**Figure 2:**
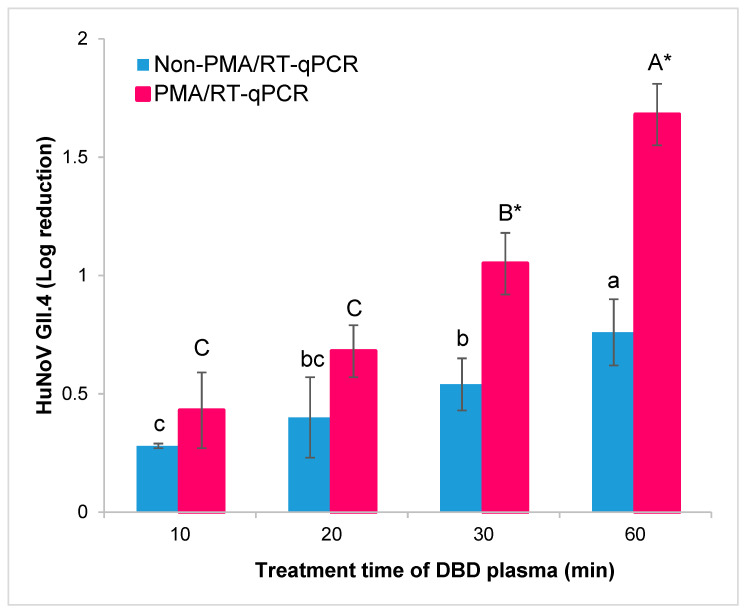
The capital letters (A–C) represent propidium monoazide (PMA) pretreated HuNoV reduction. The lowercase letters (a–c) represent non-PMA pretreated HuNoV reduction. Within each DBD plasma treatment time, log reduction means with different letters (a–c or A–C) differ significantly (*p* < 0.05) by Duncan’s multiple range test. Asterisks (*) also indicate a significant difference (*p* < 0.05) between non-PMA- and PMA-treated samples DBD plasma treated for >30 min by paired *t*-test.

Second, a comparison between PMA or non-PMA for each DBD plasma treatment time of oysters was conducted. Using the paired *t*-test, HuNoV titers were not significantly different between non-PMA- and PMA-treated samples when treated with DBD plasma until 20 min of treatment (*p* > 0.05). However, when treatment with DBD plasma occurred for 30 or 60 min, a paired *t*-test revealed that HuNoV titers were significantly different (*p* < 0.05). The differences between non-PMA- and PMA-treated HuNoV titer in oysters with different DBD plasma treatment times are as follows: 10 min (0.15 log copy number/μL, 29.21% reduction), 20 min (0.28 log copy number/μL, 47.52% reduction), 30 min (0.51 log copy number/μL, 61.10% reduction), and 60 min (0.92 log copy number/μL, 87.98% reduction). Especially at 30 min and 60 min of DBD plasma treatment, the titers of HuNoV were more significantly (*p* < 0.05) reduced in PMA- than non-PMA-treated oysters (Figure 2). These results indicate that the PMA/RT-qPCR method effectively evaluates HuNoV viability according to DBD plasma treatment time (i.e., >30 min) in oysters. Therefore, the titer of HuNoV over DBD plasma treatment can determine the D_1_-values (1-log copy number/μL and 90% log reduction) for the infectivity of HuNoV in PMA pretreatment samples.

### 3.2. Influence of DBD Plasma Treatment on HuNoV D_R_-Values in Oyster

Based on the HuNoV GII.4 survival curves generated for samples treated with varying DBD plasma treatment times, D_R_-values were obtained using the first-order kinetics model (Table 2). For the kinetics of the inactivation of microorganisms, the log-linear model’s decimal reduction time is widely accepted. The R^2^ value was 0.98, indicating that this log-linear kinetic model for HuNoV GII.4 was suitable for determining the D_R_-values. D_1_ and D_2_ values for the DBD plasma treatment were 36.5 ± 1.1 and 73.0 ± 2.3 min, respectively. Therefore, the first-order kinetics model states that the HuNoV GII.4 inactivation rate is proportional to the DBD plasma treatment time.

### 3.3. Changes in pH and Hunter Color When Using DBD Plasma Treatment in Oyster

Changes in pH and Hunter colors of fresh oysters following different treatment times of DBD plasma are shown in Table 3. No significant (*p* > 0.05) differences in pH values between the samples treated with or without DBD plasma were observed; all oyster samples were observed to have weak acid pH (approx. 5.50).

Additionally, to assess any potential mechanical color differences caused by the DBD plasma treatment, the evaluation of Hunter color “L” (black to white), “a” (green to red), and “b” (blue to yellow) values of oysters were conducted. Hunter color “L”, “a” and “b” values of oysters were not significantly (*p* > 0.05) different with or without DBD plasma treatment. These results indicate that treatment with DBD plasma did not influence the color of oyster meat.

## 4. Discussion

The DBD plasma used in this study generates plasma at low temperature and atmospheric pressure; it is called cold plasma (CP) or atmospheric pressure plasma (APP). CP or APP technology mainly includes dielectric barrier discharge (DBD) plasma, jet plasma, and corona discharge plasma [19]. CP is useful for heat-sensitive products, prevents contamination, is non-toxic, and reduces chemical agents, which is also beneficial from an environmental perspective. In particular, DBD plasma is known as a technique suitable for food sterilization because it can treat a large area for a long time stably without an electric shock [15,17]. Indeed, Yong et al. [30] showed effective sterilization of foodborne bacteria in cheese by air discharge DBD plasma for 15 min (i.e., 2.88, 3.11, and 2.26 log reductions in *E. coli*, *Salmonella typhimurium*, and *Listeria monocytogenes*, respectively).

The difference in the microbial reduction of plasma treatment has two major aspects. First, plasma treatment for microbial reduction depends primarily on the plasma difference, such as type of plasma discharge, plasma exposure type, injected gas type, electrode configuration, and frequency of applied voltage [31]. Second, it is influenced by differences in microbial species (e.g., peptidoglycan thickness, virion) or food matrix (e.g., substance state, pH) effects of the sample used for plasma treatment [17,32]. Similar to the results of this study, Ahlfeld et al. [20] reported that the inactivation of HuNoV gradually increased as the plasma treatment time increased. Ahlfeld et al. [20] reported that a 1.69 genomic equivalents/mL reduction in HuNoV GII.4 was achieved by surface micro discharge plasma (8.5 kV, 1 kHz) for 15 min. This has a greater effect than our HuNoV reduction; the main reason is that the plasma was treated in the suspension and that the microdischarge plasma used a higher output voltage than ours (1.1 kV). Bunz et al. [21] reported the plasma’s virucidal effect on some types of human adenovirus using plasma jets (60V, 60Hz, Ar 5L/min). They also speculated that the virucidal effects of plasma treatment might differ for each virus species or type. Indeed, in Bae et al. [33], murine norovirus (MNV-1), as known HuNoV’s surrogate, was inoculated into fresh meat (e.g., beef loin, pork shoulder, chicken breast) and treated with arc-plasma-based APP jets for 20 min, resulting in a high virucidal effect of approximately 2-log PFU/mL. Bae et al.’s results [33] contrast with the results of this study, which showed a 0.68 log reduction of HuNoV even with DBD plasma and PMA pretreatment. This is because the local treatment capacity of jets plasma, arc gas discharge, and food matrix is presumed. It was also estimated that the treatment of the HuNoV surrogate showed a higher HuNoV virucidal effect. However, this treatment showed a significant increase in the value of 2-thiobarbituric acid reactive substances (TBARS) in fresh meat compared to control (*p* < 0.05). Using the EMA/RT-qPCR method, Aboubakr et al. [34] indicated that a 2.6 log genomic copy of HuNoV GII.4 on steel disk surfaces and lettuce leaves was inactivated following a 5-min treatment with 2D-AICM DBD plasma. 2D-AICM DBD plasma produces singlet delta oxygen upon discharge, and these results were superior to the HuNoV virucidal effect of the current study’s DBD plasma treatment. Single delta oxygen is a fatal oxidizing agent in food and has potent cytotoxicity that can also inactivate cancer cells [35]. Therefore, it should also be noted that for the application of 2D-AICM DBD plasma to the food industry, further studies are needed to confirm that no harmful byproducts are generated by 2D-AICM DBD plasma treatment.

Intercalating dye (i.e., PMA and EMA) pretreatment prevents the amplification of capsid-damaged virus particles during RT-qPCR and allows intact capsid virus particles to be identified [32]. In Jeong et al.’s study [27], PMA and an EMA pretreatment to detect infectious HuNoV in suspensions heated at 85 °C for 1 min showed 2.93 and 2.43 log reductions, respectively. Intercalating dye pretreatment samples showed a significant decrease compared to control (*p* < 0.05); even the PMA pretreatment effect showed a more significant decrease than the EMA pretreatment effect (*p* < 0.05). Similarly, Moreno et al. [36] reported that reduction of thermally inactivated HAV in fish and shellfish treated with proteinase K, as in this study, resulted in a maximum reduction of 2.14 TCID_50_ (50% tissue culture infectious dose) log PCRU over control when pretreated with PMA + triton. PMA can strongly inhibit RT-qPCR amplification by crosslinking to accessible viral nucleic acid in damaged virions when exposed to intense visible light, but it is unclear whether it is effective for viral cells with both an intact capsid and the genome of the secondary structure [11,36]. In recent years, studies on the development of a HuNoV replication culture system derived from stem cells are being conducted to more clearly apply to the actual antiviral study of HuNoV [37]. Examples of HuNoV reproducible cultivation systems include a method to reproduce natural intestinal epithelium by generating human intestinal enteroids, a gnotobiotic pig model using porcine gastric mucins that are chemically similar to human histo-blood antigens (HBGAs), and a method of injecting HuNoV into the yolk (food reserve) of zebrafish larvae [37,38,39]. However, these methods are not yet suitable for extensive research, and the difficult process of cell culture and animal ethics problems remains, making it difficult to apply them to studies that estimate HuNoV infectivity right away. On the other hand, it is still believed that PMA/RT-qPCR analysis is a suitable indirect method to estimate a virus’s infectivity. Indeed, our study also demonstrated that when DBD plasma was used to treat oysters for 30 and 60 min, the HuNoV GII.4 viability upon PMA pretreatment was significantly (*p* < 0.05) reduced. In particular, when DBD plasma was treated for 60 min, the reduction of 0.88 log was higher than that of control; it can be expected that RT-qPCR amplified the damaged HuNoV capsid in the non-PMA sample. However, it is speculated that there was no significant difference as PMA partially penetrated the damaged virion up to the 20 min treatment time with DBD plasma, which did not sufficiently damage the capsid protein.

The inactivation of microorganisms using plasma treatment generally results in an exponential decay, depending on time [26]. In the current study, the HuNoV GII.4 survival curve in only PMA-treated oyster was fitted using the first-order kinetics model. We calculated D_1_ and D_2_-values because the dose of HuNoV required to cause an infection may be as low as 10 to 100 infection particles [40]. Park and Ha [41] indicated that the D_1_-value of MNV-1 was 21.7 min using photoplasma, which was much shorter than our D_1_-value of HuNoV (36.5 ± 1.1 min). This is because the photoplasma used in Park and Ha’s study [41] exposes the air used for plasma discharge to inner deep-UV light (30 W pressure UV-C lamp) to generate plasma and photons together, which has a strong virucidal effect. Another reason is that MNV-1, known as a surrogate for HuNoV, is less resistant to reactive species (e.g., ROS and RNS), which leads to oxidation and damage to more HuNoV capsid proteins against reactive species produced during plasma generation [3,34]. These data suggest that HuNoV GII.4 inactivation in commercial raw oysters using DBD plasma can be successfully modeled using first-order kinetics.

Meanwhile, to justify the commercializing of DBD plasma technology, the effect on raw oyster quality should be studied. In Korea, the national federation of fisheries cooperatives conducts its auction phase by measuring pH differences so that fresh raw oysters can be distributed. Changes in the pH of oysters are considered the simplest and most important indicator of a change in quality [42]. Secondarily, food products’ color is a crucial attribute that directly affects consumer perception and overall acceptability [43]. Our observed results are that, following DBD plasma treatment, the pH or Hunter color of oysters did not change significantly. Oysters contain large amounts of glycogen, which, after glycolysis, produces lactate, a substance that leads to a decrease in pH [44]. Similar to our study, Albertos et al. [29] noted that Atlantic mackerel fillet had no clear pH change (approx. 6.25–6.35). Many studies have reported that plasma treatment did not affect pH in liquid-phase foods with a buffering capacity [17,21,43]. The living tissues’ physiological activity and the possibility of the liquid emanating from the damaged tissues being used to wash away acidic compounds from the plasma treatment indicate a buffering capacity for pH on the surface [45]. However, Oehmigen et al. [46] reported that ROS and RNS produced by plasma could generate nitric acid formation or H_2_O_2_ in foods, thereby reducing the foods’ pH.

Kim et al. [47] reported that the Hunter colors did not change significantly (*p* > 0.05) when RF-driven and DBD plasma were used to treat dried laver, respectively, for 10 min. When developing a method to sterilize raw oysters, it is essential to ensure that the oysters’ color is maintained. For example, the high hydrostatic pressure that revolutionized the oyster-shucking process is known to inactivate foodborne bacteria effectively, but at pressures above 300 MPa, it has been found to change the color of oysters as a result of protein denaturation [48,49]. Taken together, treating oysters with DBD plasma for up to 1 h did not appear to affect the pH or Hunter colors. Therefore, it is considered suitable as a sterilization technology.

Application of DBD plasma treatment technology in the oyster industry can provide a nonthermal sterilization treatment that can induce effective HuNoV removal while maintaining oyster quality. Bazaka et al. [50] mentioned that plasma treatment technology prevents cross-contamination of food by preventing microbial adhesion and biofilm formation. Hence, this technology could be a breakthrough proposition to eliminate cross-contamination, especially in the oyster processing industry, where there is much manual labor. Previous studies have also demonstrated that DBD plasma treatment increases and preserves food shelf life [43].

In this study, the virucidal effect of HuNoV and simple quality indicators (e.g., pH and Hunter colors) were studied. As a limitation of this study, follow-up studies are needed to determine how DBD plasma treatment affects the oysters’ texture, nutritional properties (e.g., glycogen), and sensory properties. There is also a need to expand the research into other genotypes of HuNoV and naturally contaminated oysters. However, since there has been no study on sterilization techniques that can effectively sterilize HuNoV, a virus that causes food poisoning, while maintaining the quality of oysters, this study can be used as basic research data for DBD plasma treatment of fish and shellfish, including oysters.

## 5. Conclusions

The current study demonstrated that 1.05 and 1.68 log reductions of HuNoV in fresh oysters were achieved following nonthermal DBD plasma treatment for 30 and 60 min without a change in quality (as assessed by pH and Hunter colors). These results suggest that PMA/RT-qPCR may be useful in detecting HuNoV infectivity following DBD plasma treatment for an extended exposure time (e.g., >30–60 min). Based on first-order kinetics (R^2^ = 0.98), D_1_ (90% reduction) and D_2_ (99% reduction), following the DBD plasma treatment of oysters, were 36.5 and 73.0 min, respectively. These results could provide new hygiene information and potential approaches that should be applied to the oyster industry’s production and distribution.

## Figures and Tables

**Table 1:** Effect of dielectric barrier discharge (DBD) plasma treatment against HuNoV GII.4 inoculated oyster.

DBD Plasma Treatment (min)	Non-PMA/RT-qPCR	PMA/RT-qPCR	Before/After Using PMA to HuNoV Reduction Difference (Log Titer, % Reduction)
	Log Copy Number/µL		
Control	5.51 ± 0.02 ^a^	5.51 ± 0.04 ^a^	–
10	5.23 ± 0.01 ^b^	5.08 ± 0.16 ^b^	0.15 (29.21%)
20	5.11 ± 0.17 ^bc^	4.83 ± 0.11 ^c^	0.28 (47.52%)
30	4.97 ± 0.11 ^c^	4.46 ± 0.13 ^d^	0.51 (61.10%)
60	4.75 ± 0.14 ^d^	3.83 ± 0.13 ^e^	0.92 (87.98%)

Within the same column, HuNoV log reduction means with different letters (^a–d^ for non-PMA/RT-qPCR or ^a–e^ for PMA/RT-qPCR) differ significantly (*p* < 0.05) by Duncan’s multiple range test. The colored column also indicates a significant difference (*p* < 0.05) between non-PMA- and PMA-treated samples by paired *t*-test.

**Table 2:** Effect of DBD plasma treatment on the D_R_-values of HuNoV GII.4 with PMA reduction by first-order kinetics model in the oyster.

Enteric Microorganisms	Equation of the First-Order Kinetic Model	D_1_-Values of DBD Plasma (min)	D_2_-Values of DBD Plasma (min)	R^2^
HuNoV GII.4 with PMA	Y = −0.03x + 5.40	36.5 ± 1.1	73.0 ± 2.3	0.98

D_R_-values, decimal of log reduction time. R^2^, correlation coefficient. D = −1/slope from a plot of log copy number/µL when using DBD plasma treatment. Values, mean ± standard deviations.

**Table 3:** Changes in pH and Hunter colors when using DBD plasma treatment in oysters.

DBD Plasma Treatment (min)	pH	Hunter Colors		
		“L”	“a”	“b”
Control	5.49 ± 0.03 ^NS^	41.57 ± 0.38 ^NS^	−0.21 ± 0.06 ^NS^	12.56 ± 0.99 ^NS^
10	5.49 ± 0.09	41.80 ± 1.22	−0.24 ± 0.03	12.79 ± 1.52
20	5.48 ± 0.06	41.17 ± 1.13	−0.19 ± 0.03	13.02 ± 1.21
30	5.47 ± 0.02	40.29 ± 1.05	−0.15 ± 0.04	13.08 ± 1.43
60	5.46 ± 0.01	40.09 ± 1.63	−0.15 ± 0.09	13.43 ± 0.66

^NS^, all data were not significantly different (*p* > 0.05) according to Duncan’s multiple range test. Hunter “L” values = whiteness+, darkness–; Hunter “a” values = redness+, greenness–; Hunter “b” values = yellowness+, blueness–.
